# Novel replisome-associated proteins at cellular replication forks in EBV-transformed B lymphocytes

**DOI:** 10.1371/journal.ppat.1008228

**Published:** 2019-12-16

**Authors:** Huanzhou Xu, Ramon D. Perez, Tiffany R. Frey, Eric M. Burton, Sudha Mannemuddhu, John D. Haley, Michael T. McIntosh, Sumita Bhaduri-McIntosh

**Affiliations:** 1 Division of Infectious Disease, Department of Pediatrics, University of Florida, Gainesville, Florida, United States of America; 2 Department of Microbiology and Immunology, Stony Brook University, Stony Brook, New Yordk, Unites States of America; 3 Division of Nephrology, Dept. of Pediatrics, University of Florida, Gainesville, Florida, United States of America; 4 Department of Pathology and Stony Brook Proteomics Center, Stony Brook University, Stony Brook, New York, United States of America; 5 Child Health Research Institute, Department of Pediatrics and of Molecular Genetics and Microbiology, University of Florida, Gainesville, Florida, United States of America; 6 Division of Infectious Disease, Departments of Pediatrics and of Molecular Genetics and Microbiology, University of Florida, Gainesville, Florida, United States of America; Brigham and Women's Hospital, UNITED STATES

## Abstract

Epstein-Barr virus (EBV) is an oncogenic herpesvirus and WHO class 1 carcinogen that resides in B lymphocytes of nearly all humans. While silent in most, EBV can cause endemic Burkitt lymphoma in children and post-transplant lymphoproliferative disorders/lymphomas in immunocompromised hosts. The pathogenesis of such lymphomas is multifactorial but to a large extent depends on EBV’s ability to aggressively drive cellular DNA replication and B cell proliferation despite cell-intrinsic barriers to replication. One such barrier is oncogenic replication stress which hinders the progression of DNA replication forks. To understand how EBV successfully overcomes replication stress, we examined cellular replication forks in EBV-transformed B cells using iPOND (isolation of Proteins on Nascent DNA)-mass spectrometry and identified several cellular proteins that had not previously been linked to DNA replication. Of eight candidate replisome-associated proteins that we validated at forks in EBV-transformed cells and Burkitt lymphoma-derived cells, three zinc finger proteins (ZFPs) were upregulated early in B cells newly-infected with EBV in culture as well as expressed at high levels in EBV-infected B blasts in the blood of immunocompromised transplant recipients. Expressed highly in S- and G2-phase cells, knockdown of each ZFP resulted in stalling of proliferating cells in the S-phase, cleavage of caspase 3, and cell death. These proteins, newly-identified at replication forks of EBV-transformed and Burkitt lymphoma cells therefore contribute to cell survival and cell cycle progression, and represent novel targets for intervention of EBV-lymphomas while simultaneously offering a window into how the replication machinery may be similarly modified in other cancers.

## Author summary

Cancer cells must overcome chronic replication stress, a central barrier to DNA replication. This is true also for cancers caused by Epstein-Barr virus (EBV). To understand how EBV overcomes this barrier to successfully drive cell proliferation, we isolated proteins associated with the cellular replication machinery in EBV-transformed B lymphocytes and identified several cellular proteins that had not previously been linked to DNA replication in cancer or healthy cells. Three of these were zinc finger proteins enriched at the replication machinery in EBV-transformed and EBV-positive Burkitt lymphoma-derived cells, upregulated in newly-infected B cells, and expressed at high levels in infected B cells from transplant recipients. These zinc finger proteins also contributed towards cell proliferation, survival, and cell cycle progression. While these proteins may also contribute to DNA replication in other cancers, they simultaneously represent potential targets in EBV-cancers, some of which are difficult to treat.

## Introduction

Epstein-Barr virus post-transplant lymphoproliferative disorders/lymphomas (EBV-LPD) of B lymphocytes arises during immunosuppression that results from the use of medications aimed to prevent rejection of transplanted organs or used to treat autoimmune diseases. LPD is a serious complication following hematopoietic or organ transplantation as many recipients experience primary EBV infection or reactivate EBV during medically-imposed T cell-immunosuppression. In the absence of T cell surveillance, newly-infected B lymphocytes proliferate rapidly, often leading to LPD [1]. Therapeutic options for LPD are restricted to reduction of immunosuppression, ablation of B cells using monoclonal antibodies to CD20, and adoptive T cell therapy [1–3]–all associated with significant limitations. Reduced dosing of immunosuppressive medications places the transplanted organ at risk for rejection, global (and often long term) removal of B lymphocytes increases the risk of infectious complications, and adoptive T cell therapies are not readily available. Standard modalities such as chemotherapy, surgery, and radiation therapy may be effective in particular cases. As for antiviral strategies, these are minimally effective because anti-herpesvirus agents typically only target the lytic phase of the virus’s life cycle [4, 5].

To develop new therapeutic approaches, it is essential to delineate the molecular events that lead to B cell proliferation. EBV establishes latency in B lymphocytes in >95% of humans and while most do not develop EBV-cancers, EBV can cause immunocompromise-associated LPD in the western hemisphere and endemic Burkitt lymphoma (BL) in African children [6]. To establish latency in newly-infected B cells, EBV aggressively drives cellular DNA replication and proliferation through the activities of viral oncoproteins such as EBNA2 and LMP1; cellular oncoproteins such as Myc and STAT3 also contribute [7–9]. DNA replication is carried out in bidirectional fast-moving forks but impediments to the progress of forks are commonplace and result in fork stalling, referred to as replication stress. Such stalling activates the highly conserved replication stress response (ATR-Chk1) pathway mediated by a network of proteins to protect forks, repair DNA lesions, and accurately complete genome replication [10]. When replication is driven by oncogenes, as in EBV-cancers, such replication stress is heightened and can result in prolonged fork stalling, disassembly of the replication machinery (fork collapse), halted DNA replication, and cell death [11–13]. We, and subsequently others, have shown that EBV-transformed cells exhibit replication stress resulting in activation of the ATR-Chk1 pathway [8, 9, 14, 15]. However, we have also shown that EBV disables ATR-Chk1 signaling by impairing ATR’s ability to phosphorylate Chk1 –this viral intervention ensures that infected cells are able to travel past the intra-S phase checkpoint barrier despite replication stress [9]. How then do EBV-transformed cells combat replication stress at the cellular genome?

To understand how EBV modifies cellular replication forks to ensure B cell proliferation, we used iPOND (isolation of Proteins on Nascent DNA), a high resolution methodology to examine the composition of the replisome, a large multi-protein complex that carries out DNA replication [16, 17]. We precipitated 5-ethynyl-2′-deoxyuridine (EdU)-labeled nascent cellular DNA, i.e. replication forks, from actively proliferating EBV-transformed LCL and subjected bound proteins to mass spectrometry. After comparison to replication fork-associated proteins in activated B lymphocytes and published iPOND data from EBV-unrelated cell lines, we identified and validated eight candidate proteins at replication forks in LCL as well as in BL cells; importantly, none of these eight candidates had previously been linked to DNA replication. We further demonstrated that three zinc finger proteins (ZFPs), known or expected to bind nucleic acids, were expressed at high levels in newly-infected B cells, in S- and G2-phase LCL and BL cells in culture, and in blast-like EBV-infected B cells in the blood of transplant recipients with high EBV loads. Further, we found that these ZFPs (ZFP91, ZNF503, and ZC3H18), are important in cellular DNA replication and cell survival since their knockdown resulted in stalling of cells in the S phase of the cell cycle and cell death.

## Results

### iPOND from EBV-transformed cells (LCL) and EBV^+^ BL cells

**Fig 1:**
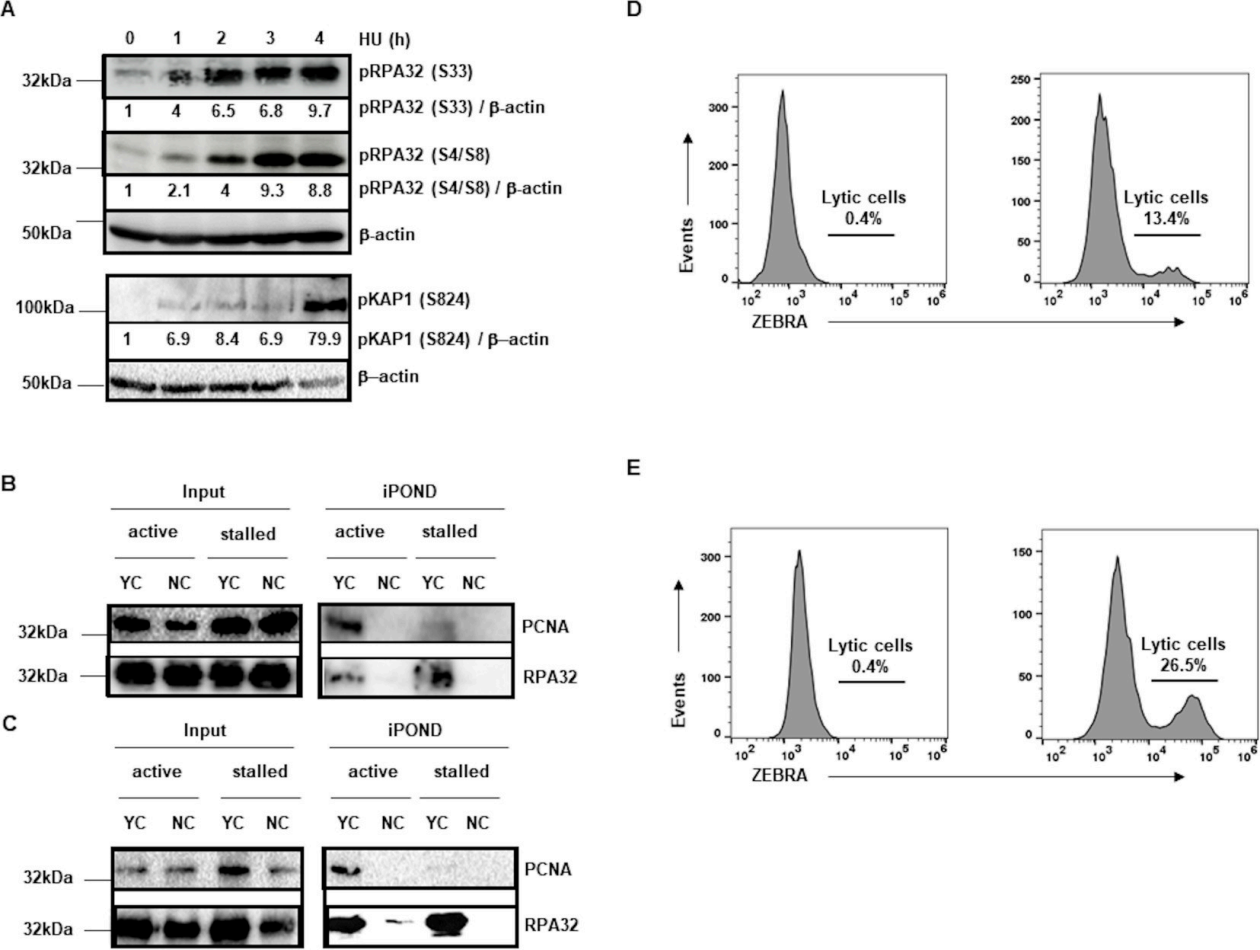
Isolation of proteins on active and stalled cellular DNA replication forks in EBV-transformed cells (lymphoblastoid cell line [LCL]) and EBV^+^ BL cells. (A) LCL were exposed to medium containing HU for different durations and lysates were subjected to immunoblotting with indicated antibodies. (B and C) LCL (B) and BL cells (C) were exposed to EdU-containing medium for 15 min prior to performing iPOND for active replication forks (active). To stall replication forks, EdU exposure was followed by exposure to HU for 2 hours followed by iPOND (stalled). Input and iPOND samples were subjected to immunoblotting with indicated antibodies. In all experiments, 0.1% of input sample was loaded. YC, yes click group, was processed with biotin-azide; NC, no click control group, was performed without biotin-azide. (D and E) LCL (D) and BL cells (E) were untreated (left-hand panels) or treated with EBV lytic cycle inducing agent sodium butyrate (right-hand panels) for 24 hours. Cells were harvested and immunostained with anti-lytic antigen ZEBRA or isotype-matched control antibodies and subjected to flow cytometry. Numbers indicate percentages of ZEBRA^+^ (lytic) cells determined by comparing with isotype-control antibody-stained cells. Experiments were performed at least thrice.

To identify proteins associated with the replication machinery not only during active DNA replication but also at recently stalled forks, we first investigated the kinetics of hydroxyurea (HU)-mediated fork stalling in EBV-transformed cells. HU inactivates cellular ribonucleotide reductase thereby inhibiting the DNA polymerase complex, resulting in stalling of replication forks [18, 19]. Such replication stress activates a complex signaling network known as the DNA damage response (DDR) whose goal is to stabilize replication forks and repair damaged DNA to ultimately complete DNA replication. The PIKK (PI3 kinase-related kinase) ATR (ATM and Rad3-related) plays a central role in replication stress-induced DDR. ATR targets RPA2 subunit (RPA32) of replication protein A to catalyze phosphorylation at serine-33 (S33), indicating newly-stalled forks [20, 21]. Another PIKK, DNA-PKcs (DNA-dependent protein kinase catalytic subunit), phosphorylates RPA32 at S4/8, indicating persistently-stalled forks [21]. We found that as early as 1 hour after exposure to HU, replication forks stalled in LCL as evidenced by phosphorylation of RPA32 at S33; this was followed by increasing phosphorylation of RPA32 at S4/8 (Fig 1A). Although stalled forks remain stable for a few hours, they eventually collapse resulting in DSBs and activation of the PIKK, ATM (ataxia telangiectasia, mutated). ATM can phosphorylate multiple targets including H2AX, BRCA1, and KRAB domain-associated protein KAP1/TRIM28 at serine 824 [22–24]. As shown in Fig 1A, substantial phosphorylation of KAP1 indicating DSBs and fork collapse, lagged until 4 hours after HU treatment. These results indicate that although fork stalling began by 1 hour, substantial fork collapse did not occur until 4 hours following exposure to HU. Based on these experiments, we determined 2 hours of exposure to HU to be adequate for causing fork stalling without significant collapse.

Next, we tested our ability to precipitate nascent DNA from active and stalled replication forks in LCL and BL cells using PCNA (proliferating cell nuclear antigen) and RPA32, both known to associate with the replication machinery [16, 21]. For iPOND, LCL and BL cells in culture were exposed briefly to EdU followed by click chemistry-mediated biotinylation of EdU in newly-replicated/nascent DNA and pulled-down using streptavidin beads. As shown in Fig 1B and 1C, iPOND effectively isolated PCNA at active replication forks only in cells in which click chemistry was used (yes click, YC); no PCNA was detected in no click (NC) samples. Moreover, when forks were stalled with HU for 2 hours, minimal amounts of PCNA were detected at stalled forks. RPA32 was also detected almost exclusively in YC samples although both at active and stalled forks in both cell lines (Fig 1B and 1C); persistent/increased detection of RPA32 at stalled forks is consistent with its recruitment to single-strand DNA (resulting from fork stalling) to protect DNA from degradation [10]. As expected, isolated nascent DNA was almost exclusively from replicating B cell genomes as less than 0.5% of LCL and BL cells at baseline (i.e. in latency) harbored actively replicating ‘lytic’ EBV genomes (Fig 1D and 1E, left-hand dotplots). These results demonstrated our ability to pull-down proteins bound specifically to newly-replicated DNA, both at active and stalled forks.

### Identification and validation of novel candidates at DNA replication forks in transformed B lymphocytes

To identify components of the replisome that were preferentially recruited to replication forks in EBV-transformed B lymphocytes compared to non-transformed but proliferating lymphocytes, we performed iPOND using LCL and B lymphocytes activated by agonists IL4 and CD40L; IL4 and CD40L were used to mimic immunologic activation of B cells [25, 26]. Proteins associated with nascent DNA were subjected to two-dimensional mass spectrometry using LC-MS/MS (mudPIT). To exclude proteins that associated with newly-synthesized but long stretches of DNA, cells were chased with thymidine for 30 minutes after EdU labeling and subjected to pull-down and mass spectrometry. Data from two sets of iPOND-MS from untreated LCL, HU-treated LCL, and activated B lymphocytes were analyzed. Proteins i) whose peptide enrichment was greater than 2 in YC compared to NC, ii) that showed no more than 1 peptide in the thymidine chase group, and iii) were not detected in the activated B lymphocyte dataset were selected. With this strategy, 99 and 116 proteins were identified at active forks (S1 Table) and stalled forks (S2 Table), respectively. When subjected to pathway analysis using IPA (Ingenuity Pathway Analysis), functional pathways that were common to the active fork and stalled fork datasets included DNA replication, damage, and repair, cell cycle, and RNA post-transcriptional modification; the top 5 identifiers in canonical pathways versus those in molecular and cellular functions for active fork and stalled fork datasets are shown in Tables 1 and 2, respectively.

**Table 1:** Biological functions and pathway analysis of proteins at active forks.

Active fork	Name
Canonical Pathways*	**EIF2 Signaling**
	**Regulation of eIF4 and p70S6K Signaling**
	**mTOR Signaling**
	**nNOS Signaling in Skeletal Muscle Cells**
	**Calcium Transport I**
Molecular and Cellular Functions*	**Protein Synthesis**
	**DNA Damage and Repair**
	**RNA Post-Transcriptional Modification**
	**Cell Cycle**
	**Cell Death and Survival**

* Top 5 pathways in each category are shown

**Table 2:** Biological functions and pathway analysis of proteins at stalled forks.

Stalled fork	Name
Canonical Pathways*	**Granzyme A Signaling**
	**Telomere Extension by Telomerase**
	**Virus Entry via Endocytic Pathways**
	**Sirtuin Signaling Pathway**
	**Acetyl-CoA Biosynthesis III (from Citrate)**
Molecular and Cellular Functions*	**RNA Post-Transcriptional Modification**
	**DNA Replication, Recombination, Repair**
	**DNA Damage and Repair**
	**Cell Cycle**
	**Cellular Assembly and Organization**

* Top 5 pathways in each category are shown

**Fig 2:**
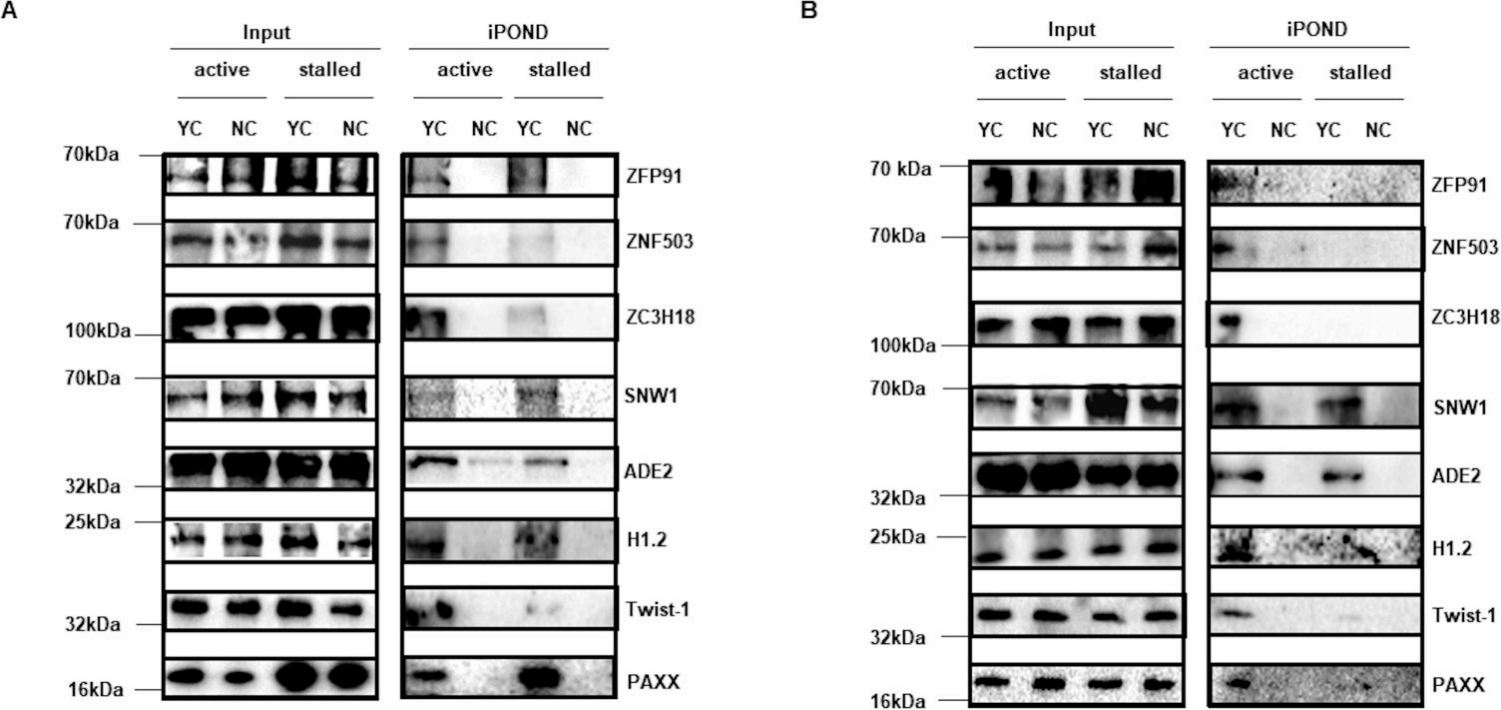
Validation of novel candidates at cellular DNA replication forks. BL cells (A) and LCL (B) cells were labeled with EdU for 15 min (active) followed by exposure to HU for 2 hours (stalled) prior to performing iPOND. Samples isolated by iPOND and 0.1% input samples were subjected to immunoblotting with indicated antibodies.

Starting with the lists of proteins at active and stalled forks, we narrowed our list of candidates to 31 proteins based on whether proteins were known to function in cell proliferation, cell cycle, DNA damage, DNA repair, DNA metabolism, nucleic acid binding, and cancer metabolism; however, proteins known to function in DNA replication or reported to be at replication forks in prior iPOND-MS studies were mostly excluded [21, 27–29]. We examined 31 candidates by iPOND followed by immunoblotting using specific antibodies and confirmed enrichment of 8 proteins at active and/or stalled forks consistently in LCL and BL cells (Fig 2A and 2B). These included 3 zinc finger proteins ZFP91, ZNF503, and ZC3H18, and 5 others including SNW1, ADE2, H1.2, Twist-1, and PAXX. Although we were able to validate SNW1 which functions in regulation of transcription and splicing, three other candidates PCBP2, hnRNPK, and Raly, also known to function in RNA biogenesis, were absent at nascent DNA when iPOND was performed in the presence of RNase A (S1 Fig); this result indicates that SNW1 was pulled-down most likely through its interaction with nascent DNA and not RNA. Thus, we validated the presence of 8 candidate proteins at replication forks in EBV-transformed cells.

### Newly-infected EBNA2^hi^ blast-like B lymphocytes express high levels of candidate ZFPs

**Fig 3:**
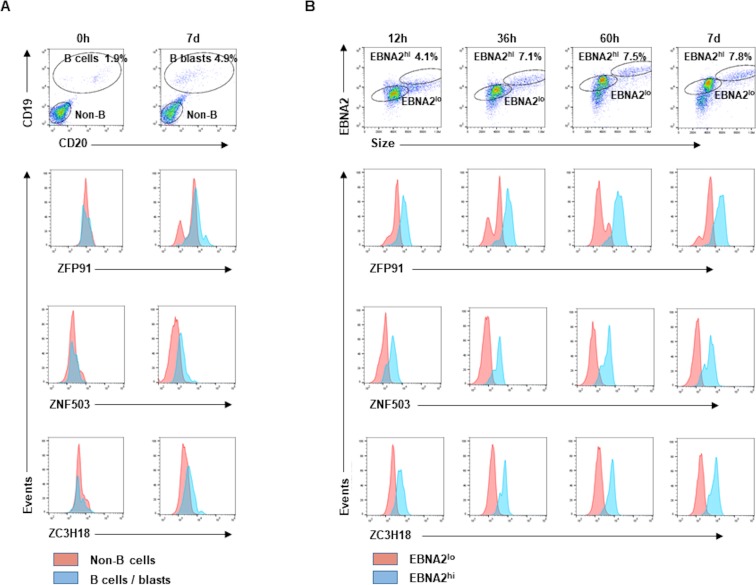
Newly-infected EBNA2^hi^ large blast-like B cells express high levels of ZFP91, ZNF503, and ZC3H18. Healthy subject-derived PBMC were stained right away (A, 0h) versus exposed to CD40L and IL4 (A, 7d) or EBV (B) and harvested for staining with antibodies to CD19 & CD20 (B cell markers; on day 7), EBNA2, and ZFPs followed by flow cytometry. Gates in top panels show Non-B & B cells/EBV^-^ B blasts (A) and EBNA2^hi^ & EBNA2^lo^ B cells (B); gates were based on isotype control staining. Lower panels show histogram overlays of ZFP expression in gated sub-populations. A representative of experiments using blood from 3 healthy subjects is shown.

Given the ability of ZFP91, ZNF503, and ZC3H18 to interact with nucleic acids and their known links to cancer [30–33], we focused on the 3 ZFPs. Because EBV is a cancer-causing virus that drives cellular DNA replication thereby causing replication stress, an obvious question is whether cells manage such oncogenic replication stress through increased expression and recruitment of proteins that may not be used under conditions of physiologic DNA replication. We therefore investigated if EBV infection altered intracellular levels of ZFPs. As control, we activated B cells using CD40L and IL4; we included this control to also ensure that the 3 ZFPs were not missed in the activated B lymphocyte-derived MS dataset due to fewer replicating cells in activated B cells compared to LCL. Seven days of activation of B lymphocytes resulted in slight increases in ZFP levels in a minority of cells compared to non-B lymphocytes; as expected, we observed an increase in the percentage of B cells by day 7 (Fig 3A). In comparison, the levels of ZFPs began to rise as early as 12 hours after infection in B cells that expressed high levels of EBNA2 compared to those expressing lower levels of EBNA2; EBNA2 is a major EBV-encoded oncogenic driver of cell proliferation in newly-infected cells [34]. Substantial increases in ZFP levels were observed in large blast-like EBNA2^hi^ cells by 60 hours (Fig 3B); such infected cells are expected to give rise to proliferating LCL. Of note, the 60-hour time-point was selected as it is known to immediately precede the earliest rounds of cell proliferation beginning around day 4 [8, 9, 35]. Thus compared to activated B cells, blast-like newly-infected B lymphocytes poised to proliferate express high levels of candidate ZFPs.

### EBV-infected B blasts in the blood of transplant recipients express high levels of candidate ZFPs

**Fig 4:**
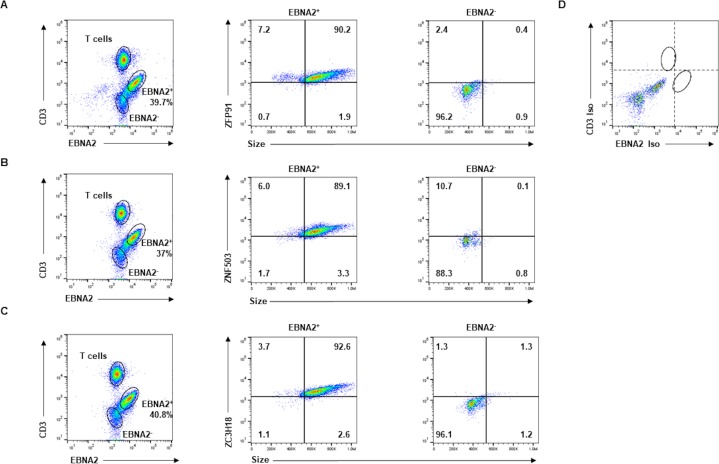
EBV-infected B blasts in the blood of transplant recipients express higher levels of ZFP91, ZNF503, ZC3H18 compared to uninfected B cells. PBMC from a transplant recipient with high blood EBV loads were stained with anti-CD3, anti-EBNA2, and anti-ZFP91 (A), anti-ZNF503 (B), or anti-ZC3H18 (C) antibodies followed by flow cytometry. Cells stained with isotype-matched control antibodies for CD3 and EBNA2 are shown in D. EBNA2^+^ and EBNA2^-^ cells (gating strategy shown in left-hand panels) were examined for ZFP expression in the middle and right-hand panels of A-C. Representative data from two transplant recipients with high EBV loads is shown.

To assess biological relevance, we asked if B lymphocytes naturally-infected with EBV in vivo also express high levels of candidate ZFPs. We examined the blood of pediatric patients who had presented with high EBV loads following solid organ transplantation. As shown in Fig 4, EBV-infected (EBNA2-positive) B lymphocytes represented a substantial fraction of PBMC; left unchecked, these cells could give rise to LPD. These EBNA2-positive, infected B lymphocytes were larger than EBNA-negative, i.e. uninfected B cells, and expressed higher levels of all 3 candidate ZFPs. Thus, both newly-infected B cells in culture and infected B lymphocytes in the blood of immunocompromised patients demonstrate elevated levels of candidate ZFPs.

### High levels of ZFPs are expressed predominantly in S and G2 phases with low levels expressed primarily in the G1 phase of the cell cycle

**Fig 5:**
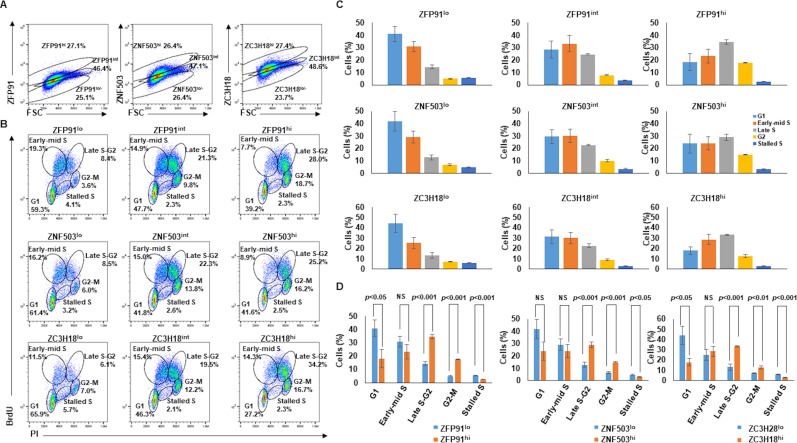
High ZFP-expressing cells are more frequently in S and G2 phases than in G1. (A and B) BL cells were labeled with BrdU for 3 hours and stained with anti-BrdU and anti-ZFP91, anti-ZNF503, or anti-ZC3H18 antibodies. Cells were divided into ZFP^hi^, ZFP^int^, and ZFP^lo^ subpopulations based on level of expression of ZFP; gating strategy is shown in A. Isotype-matched antibodies were used as control. Cell cycle distribution of ZFP^hi^, ZFP^int^, and ZFP^lo^ cells is shown in B. Representative plots are shown in A and B, with graphical representation of percent ZFP^lo^, ZFP^int^ and ZFP^hi^ cells in different phases of the cell cycle and stalled in S phase shown in C and D. Error bars, SEM; NS, not significant; experiment was performed 3 times.

With candidate ZFPs enriched at replication forks and in blast-like infected cells, and because blast-like cells are generally proliferating cells, we next examined the cell cycle distribution of cells with different levels of ZFPs. Cells were divided into 3 subpopulations: ZFP^lo^, ZFP^int^, and ZFP^hi^ representing 20–30%, 45–50%, and 20–30% of cells, respectively (Fig 5A). When each subpopulation was examined, we found that the largest fraction (~60%) of ZFP^lo^ cells were in G1 phase with fewer in S and G2 phases of the cell cycle (Fig 5B and 5C). In contrast, of ZFP^hi^ cells, 50–65% were in S and G2 phases with 27–42% in the G1 phase. ZFP^int^ cells demonstrated intermediate properties with more cells in S and G2 phases compared to ZFP^lo^ cells. We observed this significant shift towards S and G2 phases of the cell cycle with increasing levels of ZFPs in both BL cells and LCL (Fig 5C, 5D and S2 Fig). Thus, cells with high levels of candidate ZFPs tend to be in S and G2 phases of the cell cycle, consistent with enrichment of ZFPs at replication forks.

### Candidate ZFPs contribute to S phase progression and cell survival

**Fig 6:**
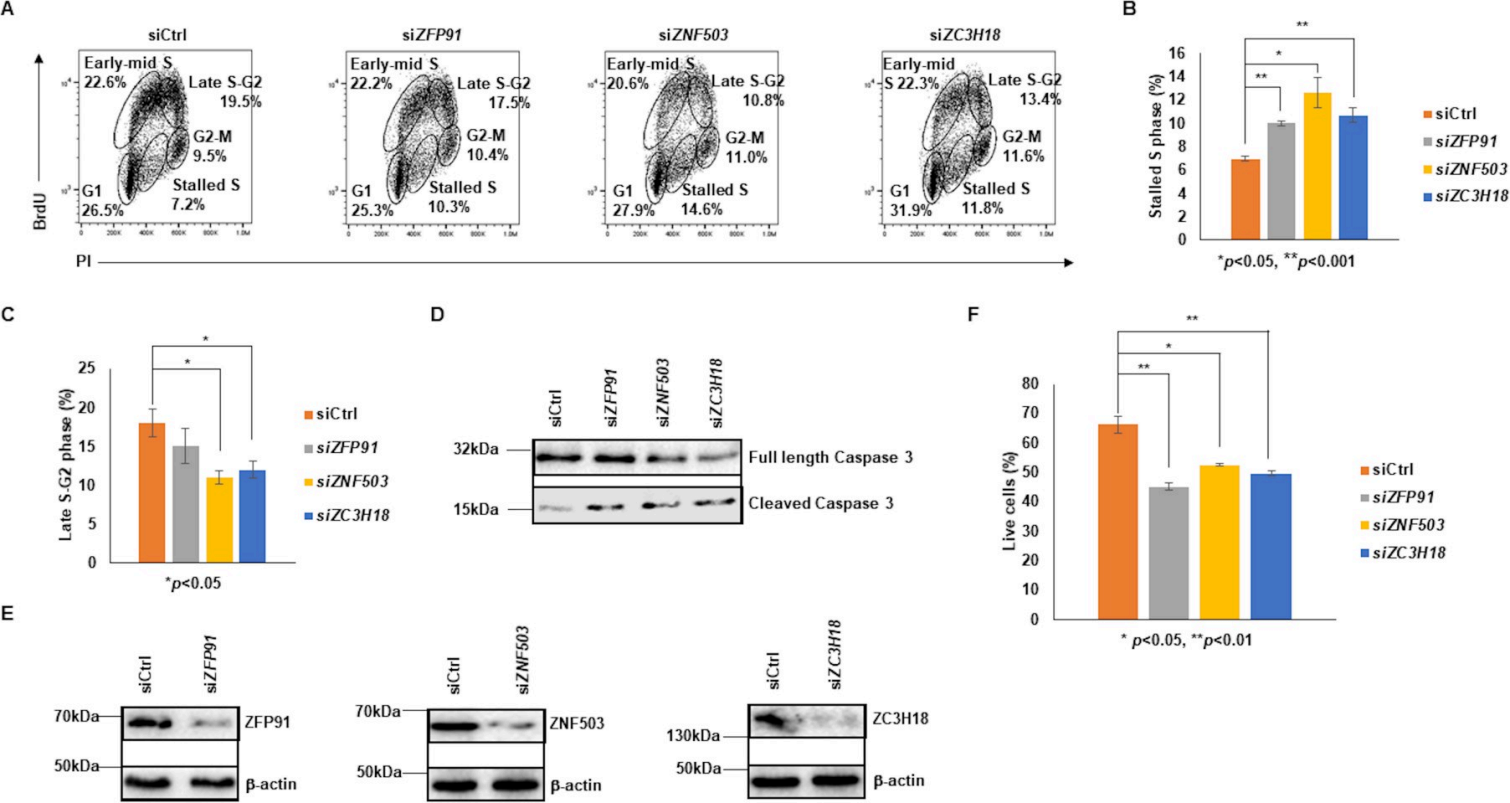
Knockdown of ZFPs results in increased stalling of cells in S phase, cleavage of caspase 3, and cell death. (A-F) BL cells were transfected with siRNA to *ZFP91*, *ZNF503* or *ZC3H18*; scrambled siRNA or mock-transfected cells were used as control. (A) After 20 hours, cells were labeled with BrdU for 3 hours and harvested for cell cycle analysis using PI, anti-BrdU antibodies, and flow cytometry. Numbers indicate percentages of cells in different phases of the cell cycle. (B and C) Percent cells that were stalled in the S phase (B) or were in late S-G2 phase (C) of the cell cycle are plotted. (D and E) Cells were harvested 20 hours after transfection and immunoblotted with indicated antibodies to determine cleavage/activation of caspase 3 (D) and knockdown efficiency of *ZFP91*, *ZNF503* and *ZC3H18* (E). (F) Cells were harvested 18 hours after transfection and percent live cells determined by PI staining and flow cytometry. Error bars in B, C, and F represent mean ± SEM of 3 experiments. All experiments were performed at least 3 times.

To determine if candidate ZFPs contribute to DNA replication, we knocked each ZFP down using siRNA. Knockdown of each ZFP resulted in significant increase in stalling of cells in the S phase of the cell cycle compared to control siRNA-transfected cells (Fig 6A and 6B, S3A and S3B Fig). A simultaneous decrease in percent cells in late S and G2 phases was also observed in BL cells (Fig 6C). Furthermore, upon knockdown of ZFPs, we observed increased cleavage of caspase 3, the major effector caspase in the cell (Fig 6D and S3C Fig) and decreased numbers of live cells (Fig 6F and S3E Fig); indeed, prolonged stalling of cells in S phase is known to induce cell death by apoptosis [13]. Similar results were obtained with a second set of siRNAs targeting ZFPs. As expected, siRNAs to ZFPs resulted in their knockdown (Fig 6E and S3D Fig). The results of these knockdown experiments are all the more relevant with them being snapshots in time and likely underestimates of the effects of ZFP knockdown.

Taken together, our findings highlight the existence of additional proteins, than previously known, at cellular replication forks. A subset, comprised of zinc finger proteins ZFP91, ZNF503, and ZC3H18, are novel candidates at the replisome primarily at active forks and contribute to the progression of cells through the S phase during DNA replication.

## Discussion

Lymphomas driven by EBV such as LPD and Burkitt lymphomas proliferate aggressively. Such cell proliferation, resulting from oncogene-mediated DNA replication, is associated with replication stress characterized by stalling of forks that can slow DNA replication. If stalled forks are not stabilized and collapsed forks not efficiently repaired, cells undergo senescence or apoptosis [13]. Indeed, our earlier work and subsequently by others, has shown that EBV-infected B lymphocytes experience replication stress as indicated by recruitment of RPA to DNA and activation of ATR [9, 14, 15]. ATR-mediated phosphorylation of Chk1 constitutes an important response to replication stress–facilitating cell cycle arrest, preventing excess origin firing, and stabilizing stalled forks [36–38]. However, we have also shown that to avoid intra-S phase arrest, EBV disables ATR-mediated phosphorylation of Chk1 through the activities of STAT3 and caspase 7 in cultured EBV-infected cells and in proliferating cells in the blood of patients with infectious mononucleosis [9]. These observations suggest that EBV uses viral or exploits pre-existing cellular mechanisms as alternative strategies to overcome barriers imposed by replication stress. We therefore examined replication forks in proliferating cells experiencing baseline (endogenous) replication stress as well as those experiencing HU-imposed added replication stress–to maximize identification of fork-associated candidates whether at active or stalled forks. As expected, analysis of biological functions confirmed that many proteins at the forks were known to be components of or associated with the replication machinery. Others were noted to function in DNA damage, DNA repair, cell cycle, and cell survival. We focused on proteins involved in DNA metabolism, cell survival, or cancer but not previously linked to DNA replication. We validated eight novel candidates at replication forks and examined three of those, all ZFPs, in further detail. Although these candidates were identified using LCL, which serves as a model for EBV-LPD, they were validated in LCL as well as in BL cells. Notably, ZFP91, ZNF503, and ZC3H18 were upregulated in EBV-infected blasts from the blood of immunocompromised recipients of solid organ transplants.

Two candidates ADE2 (or PAICS) and Twist-1 are known to be associated with human cancers. ADE2 is overexpressed in prostate, lung, and bladder cancers, reported to be an anti-apoptotic protein, and is part of the de novo purine synthesis pathway [39–42]. Twist-1, a basic helix-loop-helix transcription factor, is also overexpressed in many cancers and has roles in cancer metastasis, epithelial-mesenchymal transition, angiogenesis, chromosomal instability, resistance to platinum drugs, and evading apoptosis [43]. The third, histone H1.2, is a replication-dependent histone variant that is typically expressed during the S phase and is incorporated into chromatin during DNA replication [44, 45]. These findings support our identification of H1.2 at replication forks. As a weakly-bound variant, H1.2 is a sensitive probe for DSBs, potentially making it a valuable traveler with the replication machinery; whether it directly contributes to DNA synthesis is unclear. The fourth candidate, PAXX, was recently discovered to function in non-homologous end-joining mediated (NHEJ) DNA repair. Curiously, PAXX was dispensable for physiologic NHEJ including class switch recombination and V(D)J recombination in wild-type mice and human cells but thought instead to ensure genomic integrity under conditions of stress [46]. The fifth, SNW1, is involved in splicing and transcriptional regulation including transcription of HIV Tat and transcriptional activation by EBV EBNA2 [47–52].

Of the three ZFPs, ZFP91 and ZNF503 are C2H2 type while ZC3H18 is a C3H1 type of zinc finger protein. All three have been linked to cancer. ZFP91 is an atypical E3 ubiquitin-protein ligase that is upregulated in or promotes cancers such as acute myelogenous leukemia, prostate cancer, gastric cancer, and colon cancer [30, 53–57]. ZNF503, important for development of the brain, was found to contribute to development and progression of breast, colon, and lung cancers through transcriptional regulation of different genes [31, 58–62]. ZC3H18 is an intrinsically disordered protein that regulates RNA metabolism through transcription and RNA decay [32, 63, 64]. In colorectal cancer cell lines, depletion of ZC3H18 was found to be synthetic lethal with mutant Ras [65]. Moreover, in HEK293 cells expressing EBV LMP1, ZC3H18 activated IKK [66]. Our findings indicate that the three ZFPs are expressed at higher levels soon after EBV infection and continue to be enriched in LCL as well as BL cells transiting S and G2 phases of the cell cycle. Importantly, EBV-infected blast-like cells from the blood of transplant recipients with high EBV loads, also demonstrated high ZFPs levels. The mechanisms that regulate expression of ZFPs in EBV-infected cells are presently unclear. While EBNA2 may be a driver, it is unlikely to be the sole regulator as HH514-16 BL cells carry a deletion in the EBNA2 ORF. Another possibility is Myc as BL cells carry an Ig/Myc translocation and EBNA2^hi^ cells (LCL) express high levels of Myc. Found at replication forks, these ZFPs are important in S phase progression of cells and their survival, raising the possibility of targeting the ZFPs via synthetic lethal approaches.

To date, other iPOND studies have focused on identifying candidates at replication forks in HEK-293T cells, HeLa cells, and U2OS cells [16, 17, 67]. Our iPOND-MS is distinct in that it was designed to identify candidates in newly-transformed B lymphocytes by comparing to physiologically triggered proliferating B lymphocytes from the same host. This approach allowed us to identify proteins that were not identified in previous iPOND studies–possibly because we were able to compare between transformed and non-transformed cells. Of note also, we did not detect any EBV proteins at cellular replication forks, consistent with our observation that very few EBV-(latently) infected cells undergo lytic replication at baseline. However, in another iPOND study using Herpes Simplex virus-1-infected cells in which the virus exhibited primarily lytic replication, as expected, both cellular and viral proteins were detected at viral replication forks [28]. In Kaposi’s Sarcoma-associated Herpesvirus-infected cells, the latency protein LANA was found to recruit MCM (minichromosome maintenance protein complex) to cellular replisomes [68].

In summary, we reveal the landscape of proteins at cellular replication forks in the context of a tumor virus. While some replisome candidates may directly regulate the function of the replication machinery, others may play more supportive roles. The ultimate goal is to alleviate replication stress and thereby facilitate unhindered cell proliferation. Indeed, how chronic replication stress is alleviated remains an unresolved question even in the general cancer field as cancer cells frequently display loss of proteins that protect or repair stressed replication forks [69]. Thus, not only do these findings provide therapeutic insights for EBV-lymphomas, their relevance may extend to non-EBV cancers, as viruses frequently exploit pre-existing cellular mechanisms.

## Materials and methods

### Study subjects

Peripheral blood mononuclear cells (PBMC) were isolated from the blood of 2 patients aged 6 and 14 years with elevated circulating EBV loads following kidney transplantation. PBMC were also isolated from 3 healthy subjects who were in their early 20’s.

### Ethics statement

Blood was drawn after obtaining written informed consent from patients, or parents in case of patients who were minors. The study of human subjects was approved by the institutional review board at the University of Florida.

### Infection of PBMC

PBMC from healthy subjects were exposed to CD40L (50 ng/ml) and IL-4 (20 ng/ml) to generate EBV-negative B blasts or infected with EBV (B95-8 strain; MOI of 1–5) in the presence of 20 nM FK506, as previously described [70]. Cells were maintained in RPMI-1640 containing 10% fetal bovine serum and 1% penicillin/streptomycin and harvested at indicated time-points.

### Cell lines

EBV-transformed cell lines (LCL) were generated from healthy subjects as previously described [70]. The EBV-positive Burkitt lymphoma (BL) cell line (HH514-16) was a kind gift from Dr. George Miller at Yale University. Cell lines were maintained in RPMI-1640 containing 10% fetal bovine serum and 1% penicillin/streptomycin in the presence of 5% CO2.

### Transfection of cell lines

LCL and BL cells were subcultured at a density of 3×10^5^ to 5×10^5^/ml 24 h prior to transfection. Cells (1×10^6^) were washed once and transfected with 300 pmoles siRNA in 130 μL Ingenio solution (MIR50117, Mirus) using an Amaxa Nucleofector II (program A-024) as described previously [71]. siRNAs included *ZFP91* (Dharmacon, Cat. J-013429-09-0005 and J-013429-10-0002), *ZNF503* (Dharmacon, Cat. J-015846-17-0005 and J-015846-18-0002), *ZC3H18* (Dharmacon, Cat. J-018285-09-0005 and J-018285-17-0002), and control (Dharmacon, Cat. D001810-01-20). Cells were then seeded into pre-warmed complete media at a concentration of 5x10^5^ cells/mL and harvested as indicated.

### Isolation of proteins on nascent DNA (iPOND)

iPOND was performed as described previously with modifications [16, 17]. In brief, 1×10^8^ cells were labeled with 10 μM EdU for 15 min. For chase, EdU labeled cells were washed once with thymidine (Thy) containing medium and then incubated with 10 μM thymidine for 30 min. For stalling replication forks, 3 mM HU was directly added to EdU-containing medium for 2 hours after the 15-minute labeling step. Cells were harvested, washed once with PBS, fixed with 1% formaldehyde for 20 min at room temperature (RT), quenched with 0.125 M glycine for 5 min, permeabilized in 0.25% Triton X-100 in PBS for 30 min at RT, washed once with 0.5% BSA in PBS and once with PBS prior to performing click chemistry. For click chemistry, cells were incubated in 5 mL click reaction buffer (10 μM biotin-azide, 10 mM sodium ascorbate, 2 mM CuSO4) for 2 hours at RT. DMSO was used instead of biotin-azide in negative control (no click [NC]) groups. Cells were centrifuged (900g, 5min), washed once with 0.5% BSA in PBS and once with PBS. Nuclei were incubated in 1 ml of nuclear extraction buffer A (Cell Signaling, #7006) in the presence of 0.5 μl 1M DTT (Cell Signaling, #7006) and 1X protease inhibitor cocktail (Cell Signaling, #7012); on ice for 15min. Samples were centrifuged (2000g, 5min) at 4°C, washed once in 1 ml of buffer B (Cell Signaling, #7007) containing 0.5 μl 1M DTT and re-suspended in 200 μL buffer B. To this, 1 μL of micrococcal nuclease (Cell Signaling, #10011) was added and incubated for 20 min at 37°C with frequent mixing, followed by 10 μl of 0.5 M EDTA to stop the reaction. After centrifugation, nuclei were resuspended in cold lysis buffer (1% SDS, 50 mM Tris, pH 8.0), sonicated using a microtip sonicator at 8W with 10 sec on and 20 sec off pulses on ice three times to break nuclear membranes. Samples were centrifuged at 16,100 × g for 10 min at 4°C to remove debris and diluted with the same volume of ice-cold PBS containing protease inhibitor cocktail (Cell Signaling, #5871), and incubated with pre-washed 150 μl of streptavidin agarose beads (EMD Millipore, #69203) overnight at 4°C. Protein-DNA complexes were either analyzed by mass spectrometry or eluted with 70 μl of 2 × Laemli buffer and boiled at 95°C for 25 min, followed by immunoblotting.

### Liquid chromatography coupled with tandem mass spectrometry (LC-MS/MS)

Protein-DNA complexes on beads were washed twice with ice cold TBS, transferred to a fresh polypropylene tube, and washed once with TBS and once with cold water. Capture beads were resuspended in 100 μl 100mM ammonium bicarbonate pH 8 with 5mM DTT and heated to 90°C for 20 min. Cysteines were alkylated by addition of 10 mM iodoacetamide, incubated at RT for 30 min, and proteins digested with trypsin overnight at 37°C. Peptides were desalted on reverse phase spin tips (10 μl bed volume), dried, resuspended in 0.1% formic acid/water and subjected to LC-MS/MS.

Samples were lyophilized twice and resuspended in 20 μl 0.1% formic acid for LC-MS/MS analysis. Samples (8 μl) were injected onto a C18 trap column, washes and trap and C18 resolving column (3μ C18, 100mm x 75u ID) were placed in line and peptides eluted over a 90-minute gradient, 0–40% acetonitrile. Mass spectra were acquired on a Sciex 5600 quadrupole time-of-flight instrument (QqTOF) using an ionization voltage of 2.4kV, rolling collision energy, and a 15 second precursor ion exclusion window. MS spectra over a mass range of 400–1600 m/z triggered 20 information dependent MS/MS acquisitions over a 2.4 second cycle time with a range of 100–1200 m/z. Ion pulsing was used to increase sensitivity. The instrument was recalibrated daily using a trypsin digest of beta-galactosidase. Protein abundance was estimated by spectral counting. Peptide intensity was considered as a secondary endpoint. Proteins enriched by click groups were prioritized.

### Immunoblotting and antibodies

Immunoblotting was performed as previously described [8]. Briefly, total cell lysates were electrophoresed in 8% or 10% SDS-polyacrylamide gels, transferred to nitrocellulose membranes and stained with indicated antibodies. The following antibodies were used: rabbit anti-phospho RPA32 (S33) antibody (A300-246A-M, Bethyl Laboratories), rabbit anti-phospho RPA32 (S4/S8) antibody (A700-009, Bethyl Laboratories), rabbit anti-RPA32 antibody (A300-244A-M, Bethyl Laboratories), rabbit anti-phospho KAP1 (S824) antibody (A700-013, Bethyl Laboratories), rabbit anti-KAP1 antibody (A300-275A-M, Bethyl Laboratories), rabbit anti-PCNA antibody (A300-277A-M, Bethyl Laboratories), mouse anti-β-actin antibody (AC-15, Sigma), rabbit anti-ZFP91 antibody (A303-245A-M, Bethyl Laboratories), rabbit anti-ZNF503 antibody (A304-059A-M, Bethyl Laboratories), rabbit anti-ZC3H18 antibody (A304-682A-M, Bethyl Laboratories), rabbit anti-SNW1 antibody (PA5-29070, Thermo Fisher Scientific), rabbit anti-ADE2 antibody (A304-547A-M, Bethyl Laboratories), rabbit anti-histone H1.2 antibody (GTX122561, GeneTex), rabbit anti-C9orf142 (PAXX) antibody (A304-766A-M, Bethyl Laboratories), rabbit anti-Raly antibody (A302-070A-T, Bethyl Laboratories), mouse anti-hnRNPK antibody (sc-28380, Santa Cruz), mouse anti-PCBP2 antibody (sc-393076, Santa Cruz), mouse anti-Twist-1 antibody (NBP2-37364SS, Novus), rabbit anti-Caspase 3 antibody (GTX110543, GeneTex), rat anti-EBNA2 antibody (Clone R3, MABE8, Millipore Sigma), mouse anti-ZEBRA antibody (Clone BZ1; a kind gift from Dr. Paul Farrell at Imperial College, London), HRP-conjugated goat anti-mouse IgG(H+L) (AP308P, EMD Millipore) and HRP-conjugated goat anti-rabbit IgG(H+L) (AP307P, EMD Millipore).

### Flow cytometry

Flow cytometry was performed as previous described [9]. In short, for intracellular staining, cells were fixed with 50 μl Cytofix/Cytoperm solution (BD Biosciences, #554722) for 20 min at RT, washed twice with perm/wash buffer (BD Biosciences, #554723), incubated with primary antibody following with fluorochrome-conjugated secondary antibody. Isotype-matched antibody-stained cells were used to set cutoffs and gates during analysis. For analysis of cell cycle, cells were harvested 3 hours after incubation with 100 μM BrdU and stained as described for immunofluorescence. After incubation with primary and secondary antibodies, cells were washed and re-suspended in 150 μl of PBS containing 10 μg/ml RNase A (Thermo Scientific, #EN0531) and 20 μg/ml propidium iodide (PI, Sigma, #P4864) for 1hour at RT in the dark. Samples were then acquired using an Attune NxT Acoustic Focusing Cytometer (Invitrogen) and data were analyzed using FlowJo V10 software (Tree Star).

### Statistical analysis

*p* values were calculated by comparing the means of two groups of interest using unpaired Student t test.

## Supporting information

S1 Fig Proteins involved in RNA biogenesis PCBP2, hnRNPK, and Raly are not enriched at replication forks. LCL were labeled with EdU for 15 min (active) followed by exposure to HU for 2 hours (stalled) prior to performing iPOND in the presence of RNaseA. Samples isolated by iPOND and 0.1% input samples were subjected to immunoblotting with indicated antibodies.
(TIF)

S2 Fig High ZFP-expressing LCL tend to be in S and G2 phases than in G1. (A and B) LCL were labeled with BrdU for 3 hours and stained with anti-BrdU and anti-ZFP91, anti-ZNF503, or anti-ZC3H18 antibodies. Cells were divided into ZFP^hi^, ZFP^int^, and ZFP^lo^ subpopulations based on level of expression of ZFP; gating strategy is shown in A. Isotype-matched antibodies were used as control. Cell cycle distribution of ZFP^hi^, ZFP^int^, and ZFP^lo^ cells is shown in B. Representative plots are shown in A and B, with graphical representation of percent ZFP^lo^, ZFP^int^ and ZFP^hi^ cells in different phases of the cell cycle and stalled in S phase shown in C and D. Error bars, SEM; NS, not significant; experiment was performed 3 times.
(TIF)

S3 Fig Knockdown of ZFPs results in increased stalling of cells in S phase, cleavage of caspase 3, and death of LCL. (A-E) LCL were transfected with siRNA to *ZFP91*, *ZNF503* or *ZC3H18*; scrambled siRNA or mock-transfected cells were used as control. (A) After 20 hours, cells were labeled with BrdU for 3 hours and harvested for cell cycle analysis using PI, anti-BrdU antibodies, and flow cytometry. Numbers indicate percentages of cells in different phases of the cell cycle. (B) Percent cells that were stalled in the S phase of the cell cycle are plotted. (C and D) Cells were harvested 20 hours after transfection and immunoblotted with indicated antibodies to determine cleavage/activation of caspase 3 (C) and knockdown efficiency of *ZFP91*, *ZNF503* and *ZC3H18* (D). (E) Cells were harvested 18 hours after transfection and percent live cells determined by PI staining and flow cytometry. Error bars in B and E represent mean ± SEM of 3 experiments. All experiments were performed at least 3 times.
(TIF)

S1 Table Proteins at active forks. (PDF)

S2 Table Proteins at stalled forks. (PDF)
