# Supplementary figures and images for: Novel replisome-associated proteins at cellular replication forks in EBV-transformed B lymphocytes
Source: PLoS Pathog. 2019 Dec 16;15(12):e1008228. doi: 10.1371/journal.ppat.1008228 (PMC6936862; doi:10.1371/journal.ppat.1008228)

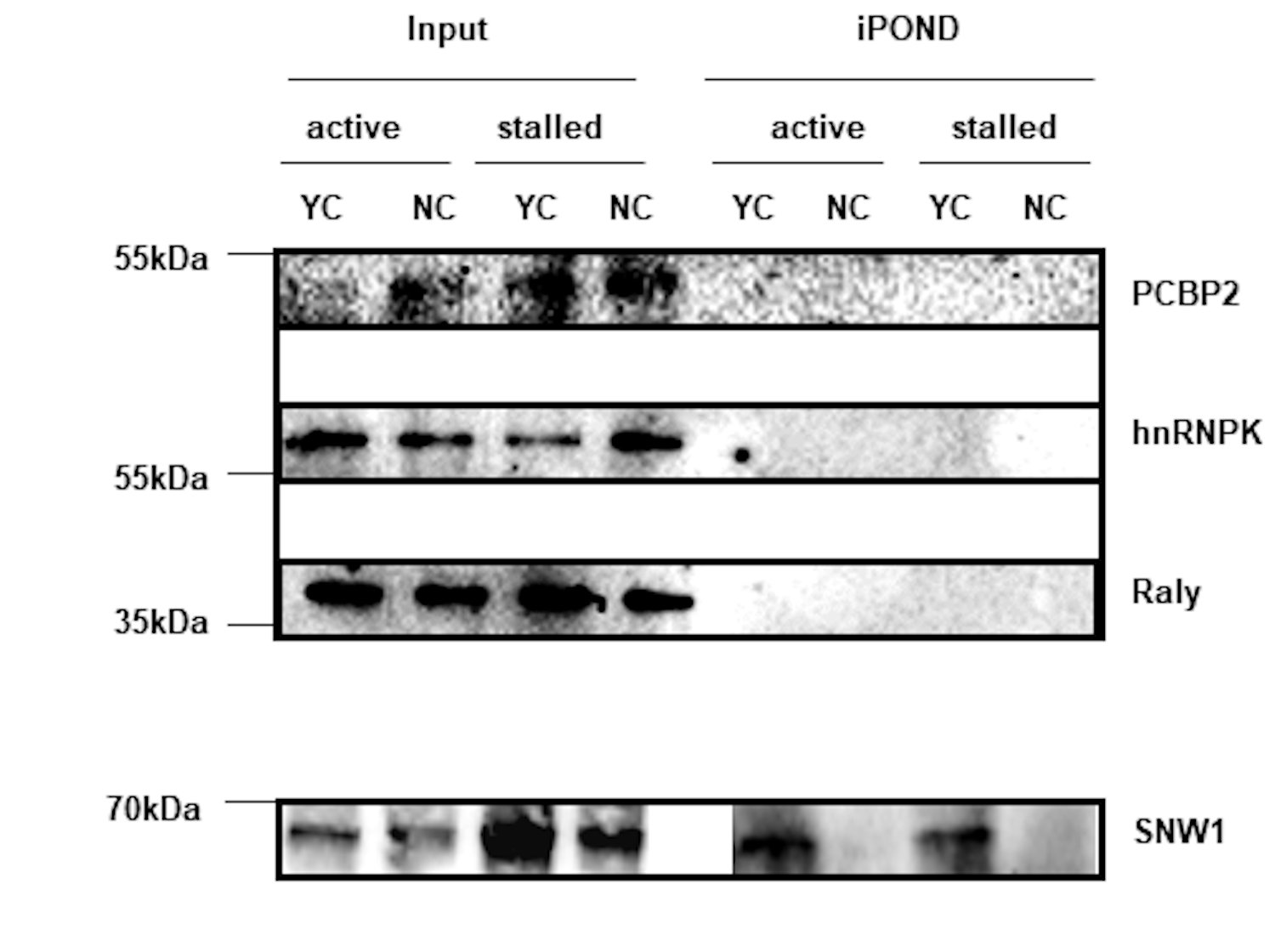

Supplement: S1 Fig — LCL were labeled with EdU for 15 min (active) followed by exposure to HU for 2 hours (stalled) prior to performing iPOND in the presence of RNaseA. Samples isolated by iPOND and 0.1% input samples were subjected to immunoblotting with indicated antibodies. (TIF) [file ppat.1008228.s001.tif]

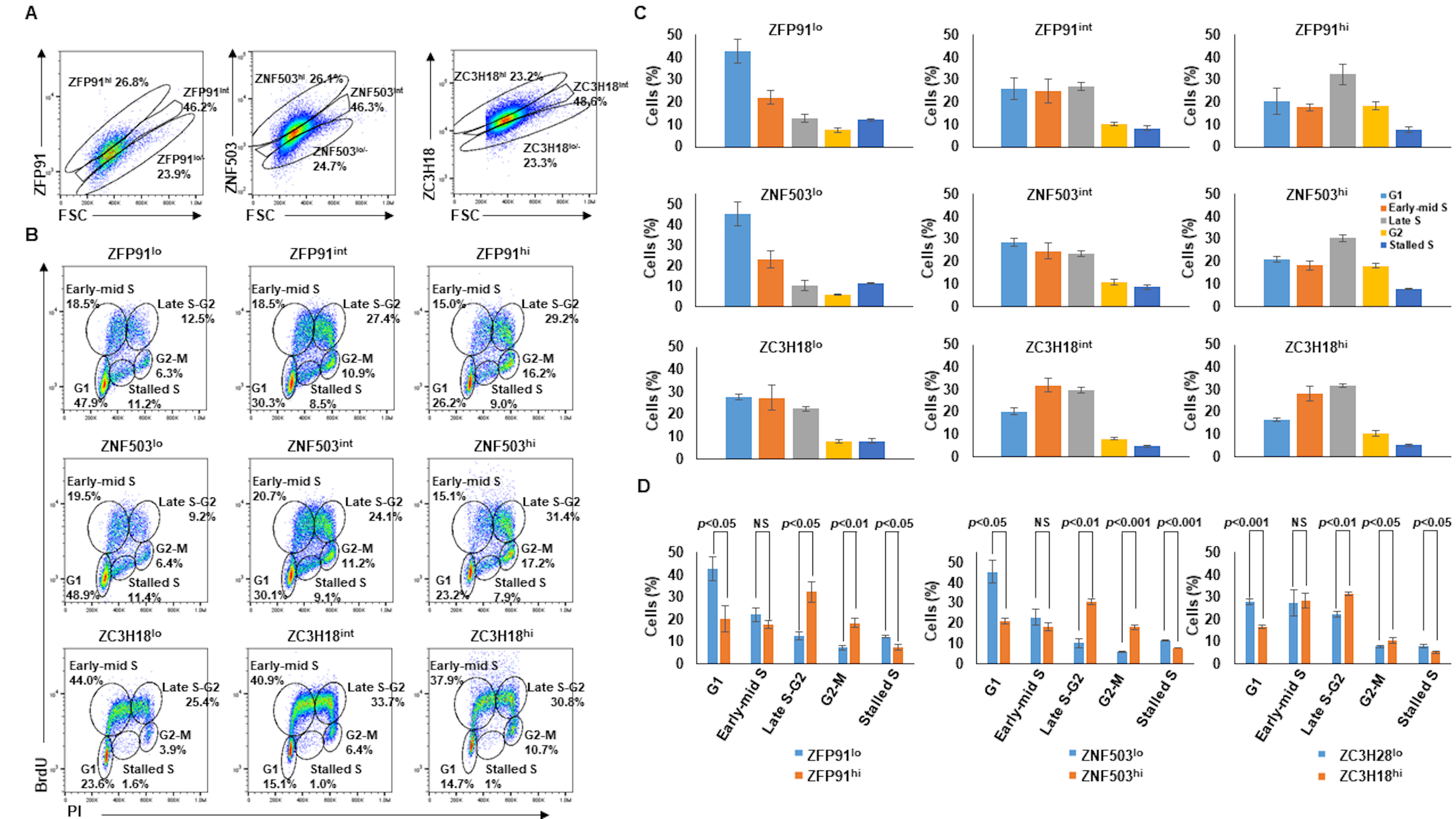

Supplement: S2 Fig — (A and B) LCL were labeled with BrdU for 3 hours and stained with anti-BrdU and anti-ZFP91, anti-ZNF503, or anti-ZC3H18 antibodies. Cells were divided into ZFPhi, ZFPint, and ZFPlo subpopulations based on level of expression of ZFP; gating strategy is shown in A. Isotype-matched antibodies were used as control. Cell cycle distribution of ZFPhi, ZFPint, and ZFPlo cells is shown in B. Representative plots are shown in A and B, with graphical representation of percent ZFPlo, ZFPint and ZFPhi cells in different phases of the cell cycle and stalled in S phase shown in C and D. Error bars, SEM; NS, not significant; experiment was performed 3 times. (TIF) [file ppat.1008228.s002.tif]

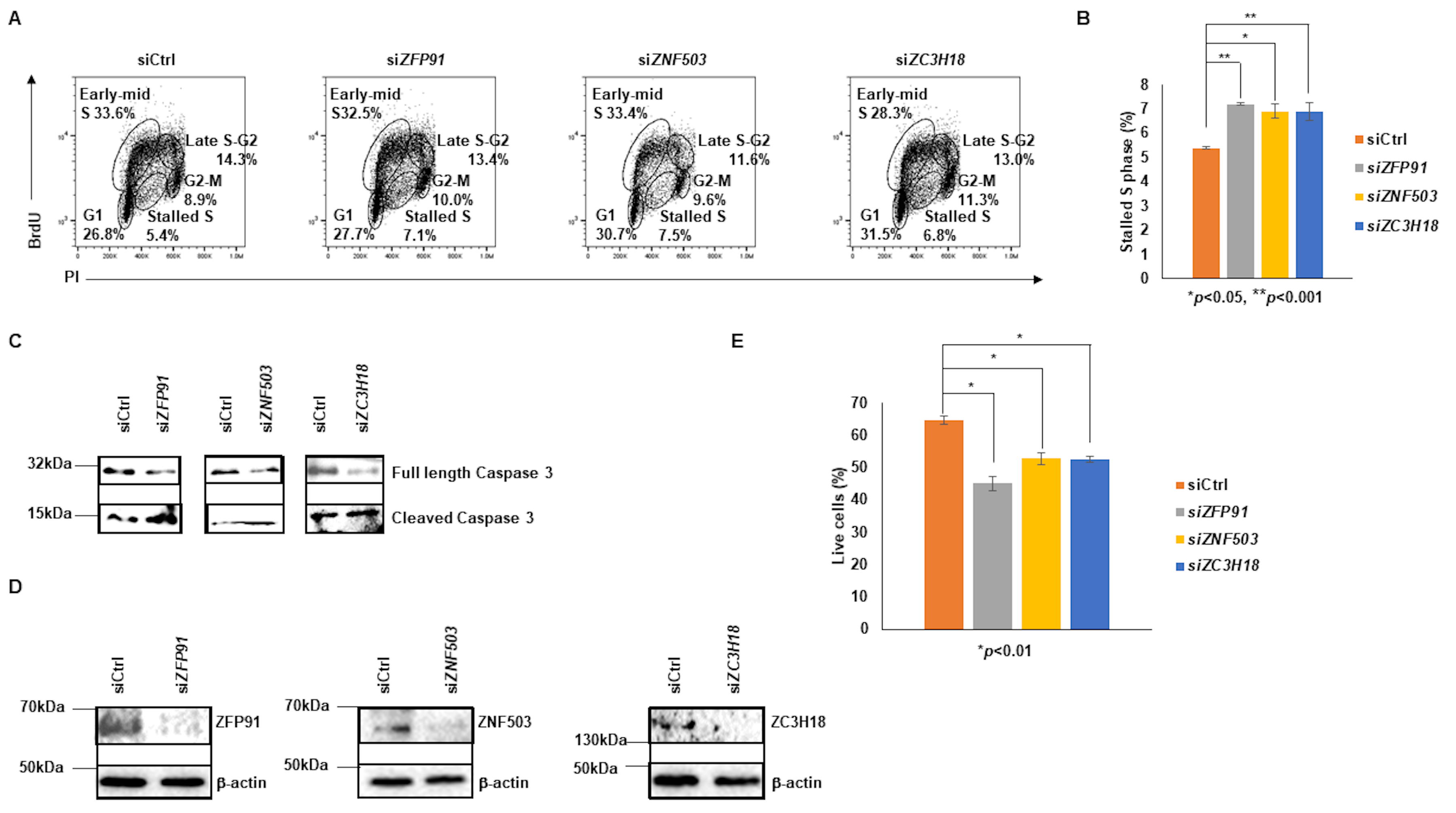

Supplement: S3 Fig — (A-E) LCL were transfected with siRNA to ZFP91, ZNF503 or ZC3H18; scrambled siRNA or mock-transfected cells were used as control. (A) After 20 hours, cells were labeled with BrdU for 3 hours and harvested for cell cycle analysis using PI, anti-BrdU antibodies, and flow cytometry. Numbers indicate percentages of cells in different phases of the cell cycle. (B) Percent cells that were stalled in the S phase of the cell cycle are plotted. (C and D) Cells were harvested 20 hours after transfection and immunoblotted with indicated antibodies to determine cleavage/activation of caspase 3 (C) and knockdown efficiency of ZFP91, ZNF503 and ZC3H18 (D). (E) Cells were harvested 18 hours after transfection and percent live cells determined by PI staining and flow cytometry. Error bars in B and E represent mean ± SEM of 3 experiments. All experiments were performed at least 3 times. (TIF) [file ppat.1008228.s003.tif]
