# Supplementary material for: Novel replisome-associated proteins at cellular replication forks in EBV-transformed B lymphocytes
Source: PLoS Pathog. 2019 Dec 16;15(12):e1008228. doi: 10.1371/journal.ppat.1008228 (PMC6936862; doi:10.1371/journal.ppat.1008228)
Supplement: S1 Table — (PDF) [file ppat.1008228.s004.pdf]

Supplemental Table 1. Proteins at active forks

UniprotKB Accession	Protein	Description
O75818	RPP40	Ribonuclease P protein subunit p40
P29692	EF1D	Elongation factor 1-delta
Q5QPL9	RALY	RNA-binding protein Raly
Q86YZ3	HORN	Hornerin
Q5CZC0	FSIP2	Fibrous sheath-interacting protein 2
Q71UI9	H2AV	Histone H2A.V
P62266	RS23	40S ribosomal protein S23
Q96E39	RMXL1	RNA binding motif protein, X-linked-like-1
Q9UMD9	COHA1	Collagen alpha-1(XVII) chain
J3KN10	PI4KA	Phosphatidylinositol 4-kinase alpha
P22234	PUR6	Multifunctional protein ADE2
P33993	MCM7	DNA replication licensing factor MCM7
Q14966	ZN638	Zinc finger protein 638
B1AKP7	B1AKP7	TAR DNA-binding protein 43 {ECO:0000313
Q9Y265	RUVB1	RuvB-like 1
P12755	SKI	Ski oncogene
Q01484	ANK2	Ankyrin-2
Q14204	DYHC1	Cytoplasmic dynein 1 heavy chain 1
Q00839	HNRPU	Heterogeneous nuclear ribonucleoprotein U
P48634	PRC2A	Protein PRRC2A
O94906	PRP6	Pre-mRNA-processing factor 6
Q96RS2	RPSA	40S ribosomal protein SA
Q9C0A1	ZFHX2	Zinc finger homeobox protein 2
Q8NDT2	RB15B	Putative RNA-binding protein 15B
Q8NFC6	BD1L1	Biorientation of chromosomes in cell division protein 1-like 1 {ECO:0000305}
Q96P50	ACAP3	Arf-GAP with coiled-coil, ANK repeat and PH domain-containing protein 3
Q7L311	ARMX2	Armadillo repeat-containing X-linked protein 2
B4DTC3	B4DTC3	cDNA FLJ54150, highly similar to Heterogeneous nuclear ribonucleoprotein D0
Q00975	CAC1B	Voltage-dependent N-type calcium channel subunit alpha-1B
P04632	CPNS1	Calpain small subunit 1
Q6PD62	CTR9	RNA polymerase-associated protein CTR9 homolog
Q14152	EIF3A	Eukaryotic translation initiation factor 3 subunit A
G3V3A4	G3V3A4	SNW domain-containing protein 1
G8JLB6	HNRNPH1	Heterogeneous nuclear ribonucleoprotein H
P41091	IF2G	Eukaryotic translation initiation factor 2 subunit 3
Q92794	KAT6A	Histone acetyltransferase KAT6A
Q86V48	LUZP1	Leucine zipper protein 1
Q08J23	NSUN2	tRNA (cytosine(34)-C(5))-methyltransferase
Q9BUH6	C9orf142	Protein PAXX
Q7Z6K3	PTAR1	Protein prenyltransferase alpha subunit repeat-containing protein 1
Q5T8U3	RPL7A	60S ribosomal protein L7a
Q8N5L9	RPS2	Ribosomal protein S2
O60902	SHOX2	Short stature homeobox protein 2
O15417	TNC18	Trinucleotide repeat-containing gene 18 protein

P55209	NP1L1	Nucleosome assembly protein 1-like 1
A0A024RAE6	CDC42	Cell division cycle 42 (GTP binding protein, 25kDa), isoform CRA_c
Q8IZP0	ABI1	Abl interactor 1
P28838	AMPL	Cytosol aminopeptidase
P16615	AT2A2	Sarcoplasmic/endoplasmic reticulum calcium ATPase 2
Q9UKP4	ATS7	A disintegrin and metalloproteinase with thrombospondin motifs 7
B3KUD6	B3KUD6	cDNA FLJ39634 fis, clone SMINT2002689, highly similar to SMOOTHELIN
B4DZP8	B4DZP8	cDNA FLJ61502
B4DZS2	B4DZS2	cDNA FLJ61505, weakly similar to Trichohyalin
B9A031	SERF2	Small EDRK-rich factor 2
O95180	CAC1H	Voltage-dependent T-type calcium channel subunit alpha-1H
A6NKD9	CC85C	Coiled-coil domain-containing protein 85C
Q9HC77	CENPJ	Centromere protein J
Q15003	CND2	Condensin complex subunit 2
Q13616	CUL1	Cullin-1
P39880	CUX1	Homeobox protein cut-like 1
O00571	DDX3X	ATP-dependent RNA helicase DDX3X
P21802	FGFR2	Fibroblast growth factor receptor 2
Q5VW38	GP107	Protein GPR107
H0YES8	RPS3	40S ribosomal protein S3
H3BQK9	MACF1	Microtubule-actin cross-linking factor 1, isoforms 1/2/3/5
P14866	HNRPL	Heterogeneous nuclear ribonucleoprotein L
Q6IS14	IF5AL	Eukaryotic translation initiation factor 5A-1-like
P52333	JAK3	Tyrosine-protein kinase JAK3
P19012	K1C15	Keratin, type I cytoskeletal 15
P31323	KAP3	cAMP-dependent protein kinase type II-beta regulatory subunit
O14495	LPP3	Phospholipid phosphatase 3
Q6F5E8	LR16C	Capping protein, Arp2/3 and myosin-I linker protein 2
O15021	MAST4	Microtubule-associated serine/threonine-protein kinase 4
Q149M9	NWD1	NACHT domain- and WD repeat-containing protein 1
Q8TAD7	OCC1	Overexpressed in colon carcinoma 1 protein
O75665	OFD1	Oral-facial-digital syndrome 1 protein
Q3SYA9	P12L1	Putative POM121-like protein 1
Q9Y5H2	PCDGB	Protocadherin gamma-A11
Q4LE60	TNPO2	TNPO2 variant protein
Q59GV0	PCCB	Propionyl Coenzyme A carboxylase, beta polypeptide variant
Q9BQ04	RBM4B	RNA-binding protein 4B
Q02543	RL18A	60S ribosomal protein L18a
O75526	RMXL2	RNA-binding motif protein, X-linked-like-2
Q8NCN4	RN169	E3 ubiquitin-protein ligase RNF169
P25398	RS12	40S ribosomal protein S12
O75533	SF3B1	Splicing factor 3B subunit 1
O75182	SIN3B	Paired amphipathic helix protein Sin3b
Q13884	SNTB1	Beta-1-syntrophin
Q9BXP5	SRRT	Serrate RNA effector molecule homolog
Q9Y2I9	TBC30	TBC1 domain family member 30
P35590	TIE1	Tyrosine-protein kinase receptor Tie-1

Q8TDI7	TMC2	Transmembrane channel-like protein 2
O75643	U520	U5 small nuclear ribonucleoprotein 200 kDa helicase
Q96JP5	ZFP91	E3 ubiquitin-protein ligase ZFP91
P27695	APEX1	DNA-(apurinic or apyrimidinic site) lyase
Q86UE4	LYRIC	Protein LYRIC
O75185	AT2C2	Calcium-transporting ATPase type 2C member 2
P39019	RS19	40S ribosomal protein S19
Q8NDV7	TNR6A	Trinucleotide repeat-containing gene 6A protein
