# Supplementary material for: Novel replisome-associated proteins at cellular replication forks in EBV-transformed B lymphocytes
Source: PLoS Pathog. 2019 Dec 16;15(12):e1008228. doi: 10.1371/journal.ppat.1008228 (PMC6936862; doi:10.1371/journal.ppat.1008228)
Supplement: S2 Table — (PDF) [file ppat.1008228.s005.pdf]

Supplemental Table 2. Proteins at stalled forks

UniprotKB Accession	Protein	Description
O75818	RPP40	Ribonuclease P protein subunit p40
P16403	H12	Histone H1.2
Q99880	H2B1L	Histone H2B type 1-L
P04264	K2C1	Keratin, type II cytoskeletal 1
O75445	USH2A	Usherin
P09651	ROA1	Heterogeneous nuclear ribonucleoprotein A1
P13645	K1C10	Keratin, type I cytoskeletal 10
B4DUX3	B4DUX3	cDNA FLJ54165
Q12791	KCMA1	Calcium-activated potassium channel subunit alpha-1
P36578	RL4	60S ribosomal protein L4
P78527	PRKDC	DNA-dependent protein kinase catalytic subunit
B3KMI9	B3KMI9	cDNA FLJ11140 fis, clone PLACE1006488, highly similar to Signal recognition particle 68 kDa protein
P23396	RS3	40S ribosomal protein S3
O75179	ANR17	Ankyrin repeat domain-containing protein 17
B3KTP9	B3KTP9	cDNA FLJ38578 fis, clone HCHON2007674, highly similar to NUCLEOLIN
P05997	CO5A2	Collagen alpha-2(V) chain
P35637	FUS	RNA-binding protein FUS
Q8NG19	Q8NG19	Multi-functional protein MFP
P38159	RBMX	RNA-binding motif protein, X chromosome
P15822	ZEP1	Zinc finger protein 40
Q96F45	ZN503	Zinc finger protein 503
O14497	ARI1A	AT-rich interactive domain-containing protein 1A
P21333	FLNA	Filamin-A
O95843	GUC1C	Guanylyl cyclase-activating protein 3
J3KNJ7	J3KNJ7	Methyltransferase-like protein 4
J3KPC8	J3KPC8	Serine/threonine-protein kinase SIK3
O15230	LAMA5	Laminin subunit alpha-5
Q9Y6V0	PCLO	Protein piccolo
Q9Y520	PRC2C	Protein PRRC2C
Q4W5T8	Q4W5T8	Putative uncharacterized protein PKP4
Q6IBR0	Q6IBR0	Dolichyl-diphosphooligosaccharide--protein glycosyltransferase subunit 1
A4UGR9	XIRP2	Xin actin-binding repeat-containing protein 2
P51825	AFF1	AF4/FMR2 family member 1
Q8IVF2	AHNK2	Protein AHNAK2
B2R959	B2R959	cDNA, FLJ94229, highly similar to Homo sapiens heterogeneous nuclear ribonucleoprotein L (HNRPL),mRNA
Q9ULD4	BRPF3	Bromodomain and PHD finger-containing protein 3
P59037	CU084	Putative uncharacterized protein encoded by LINC00313
E7BWR6	E7BWR6	KIR3DL2
H7C3B5	H7C3B5	Genetic suppressor element 1
O15131	IMA6	Importin subunit alpha-6
Q6P2Q9	PRP8	Pre-mRNA-processing-splicing factor 8
Q9P258	RCC2	Protein RCC2
Q63HN8	RN213	E3 ubiquitin-protein ligase RNF213
Q9Y2L1	RRP44	Exosome complex exonuclease RRP44

O75533	SF3B1	Splicing factor 3B subunit 1
A6NL88	SHSA7	Protein shisa-7
Q15554	TERF2	Telomeric repeat-binding factor 2
Q8NI27	THOC2	THO complex subunit 2
Q13263	TIF1B	Transcription intermediary factor 1-beta
Q15672	TWST1	Twist-related protein 1
Q70CQ4	UBP31	Ubiquitin carboxyl-terminal hydrolase 31
Q86VM9	ZCH18	Zinc finger CCCH domain-containing protein 18
A0A087X2D0	A0A087X2D0	Serine/arginine-rich-splicing factor 3
A6NLN1	A6NLN1	Polypyrimidine tract binding protein 1, isoform CRA_b
A8KAP3	A8KAP3	cDNA FLJ78483, highly similar to Homo sapiens elongation factor Tu GTP binding domain containing 2 (EFTUD2), mRNA
P30532	ACHA5	Neuronal acetylcholine receptor subunit alpha-5
Q9UKV3	ACINU	Apoptotic chromatin condensation inducer in the nucleus
P53396	ACLY	ATP-citrate synthase
O00203	AP3B1	AP-3 complex subunit beta-1
Q8IZT6	ASPM	Abnormal spindle-like microcephaly-associated protein
B3KX96	B3KX96	cDNA FLJ45003 fis, clone BRAWH3011623, highly similar to Heterogeneous nuclear ribonucleoproteins C
B8K284	B8K284	CBL-B variant
C9JT67	C9JT67	Coiled-coil domain-containing protein 144A
Q4G0X9	CCD40	Coiled-coil domain-containing protein 40
P13942	COBA2	Collagen alpha-2(XI) chain
D3DV25	D3DV25	RAR-related orphan receptor C, isoform CRA_a
O00571	DDX3X	ATP-dependent RNA helicase DDX3X
P17844	DDX5	Probable ATP-dependent RNA helicase DDX5
P35659	DEK	Protein DEK
P50570	DYN2	Dynamin-2
P29322	EPHA8	Ephrin type-A receptor 8
Q86VR2	F134C	Protein FAM134C
Q12946	FOXF1	Forkhead box protein F1
G3V5Q1	G3V5Q1	DNA-(apurinic or apyrimidinic site) lyase
G8JLB6	G8JLB6	Heterogeneous nuclear ribonucleoprotein H
Q92538	GBF1	Golgi-specific brefeldin A-resistance guanine nucleotide exchange factor 1
Q04446	GLGB	1,4-alpha-glucan-branching enzyme
H0Y8C3	H0Y8C3	Mitochondrial carrier homolog 1
H0YJF8	H0YJF8	Sorting nexin-6
H0YNX3	H0YNX3	Mortality factor 4-like protein 1
P22492	H1T	Histone H1t
H7BZ52	H7BZ52	Zinc finger SWIM domain-containing protein 8
O43593	HAIR	Lysine-specific demethylase hairless
P31942	HNRH3	Heterogeneous nuclear ribonucleoprotein H3
P20701	ITAL	Integrin alpha-L
Q9ULL0	K1210	Uncharacterized protein KIAA1210
Q6NV74	K121L	Uncharacterized protein KIAA1211-like
Q14678	KANK1	KN motif and ankyrin repeat domain-containing protein 1
Q9UMN6	KMT2B	Histone-lysine N-methyltransferase 2B
P05455	LA	Lupus La protein
Q96B70	LENG9	Leukocyte receptor cluster member 9

Q8IVH8	M4K3	Mitogen-activated protein kinase kinase kinase kinase 3
Q13724	MOGS	Mannosyl-oligosaccharide glucosidase
Q96DH6	MSI2H	RNA-binding protein Musashi homolog 2
P20929	NEBU	Nebulin
Q9H8H0	NOL11	Nucleolar protein 11
P48552	NRIP1	Nuclear receptor-interacting protein 1
Q96RV3	PCX1	Pecanex-like protein 1
Q9NP80	PLPL8	Calcium-independent phospholipase A2-gamma
Q4LE60	Q4LE60	TNPO2 variant protein
Q9NWC0	Q9NWC0	cDNA FLJ10141 fis, clone HEMBA1003199
Q7Z5J4	RAI1	Retinoic acid-induced protein 1
Q96PK6	RBM14	RNA-binding protein 14
P35251	RFC1	Replication factor C subunit 1
P15880	RS2	40S ribosomal protein S2
Q96T23	RSF1	Remodeling and spacing factor 1
Q15393	SF3B3	Splicing factor 3B subunit 3
Q9BVQ7	SPA5L	Spermatogenesis-associated protein 5-like protein 1
Q9NRC6	SPTN5	Spectrin beta chain, non-erythrocytic 5
Q6UXY8	TMC5	Transmembrane channel-like protein 5
Q8N9V7	TOPZ1	Testis- and ovary-specific PAZ domain-containing protein 1
Q8TD43	TRPM4	Transient receptor potential cation channel subfamily M member 4
Q8IWX7	UN45B	Protein unc-45 homolog B
Q5THJ4	VP13D	Vacuolar protein sorting-associated protein 13D
Q9UF83	YM012	Uncharacterized protein DKFZp434B061
Q14966	ZN638	Zinc finger protein 638
